# Draft genome sequence of *Streptococcus gallolyticus* AAM02 isolated from traditional fermented milk in Bangladesh

**DOI:** 10.1128/mra.01389-25

**Published:** 2026-03-30

**Authors:** Md. Fakruddin, Md. Abu Bakar Karim, Samara Aliya Chowdhury, Abeda Sultana, Sharmin Zaman Emon, Md. Latiful Bari, Md. Arafat Al Mamun

**Affiliations:** 1 Department of Biochemistry and Microbiology, North South University 54495, Dhaka, Bangladesh; 2 Department of Anatomy, Dhaka Medical College 54495, Dhaka, Bangladesh; 3 Centre for Advanced Research in Sciences, University of Dhaka 429264, Dhaka, Bangladesh; University of Maryland School of Medicine, Baltimore, Maryland, USA

**Keywords:** genomes, Bangladesh, fermented Milk

## Abstract

We announce the draft genome sequence of *Streptococcus gallolyticus* strain AAM02, isolated from traditional fermented milk (doi) collected in Bogura, Bangladesh (GPS: 24.85°N, 89.37°E) in 2023. The genome provides insights into its genetic adaptations to the fermented milk environment and metabolic characteristics relevant to fermentation.

## ANNOUNCEMENT

*Streptococcus gallolyticus* is a gram-positive, facultative anaerobic lactic acid bacterium found in traditional fermented foods. Strain AAM02 was isolated from fermented milk samples cultured on MRS agar under microaerophilic conditions at 37°C. Preliminary identification was performed by 16S rRNA gene sequencing (primers: 27F/1492R; deposited in GenBank under accession PQ123456). For whole-genome sequencing, the strain was grown on M17 agar at 37°C overnight. Genomic DNA was extracted using the Qiagen DNeasy Blood & Tissue Kit without shearing or size selection. Libraries were prepared with the Nextera DNA Flex Library Preparation Kit, and paired-end sequencing (2 × 150 bp) was performed on an Illumina NovaSeq 6000 at icddr,b Genome Center, Bangladesh. A total of 2,856,432 reads were generated, providing ~230× coverage. Raw reads were quality-checked with FastQC v0.11.9 (1) and trimmed with Trimmomatic v0.39 (2). Assembly was performed using SPAdes v4.1.0 (3), yielding 21 contigs (N50 = 263,119 bp; N90 = 59,687 bp). The genome size is 1,834,679 bp with 37.62% G+C content. Taxonomic identification was confirmed with Kraken2 v2.1.3 (4). Annotation was performed using the NCBI Prokaryotic Genome Annotation Pipeline, identifying 1,853 coding sequences (1,298 with functional assignments, 555 hypothetical proteins), 6 rRNA genes, 52 tRNA genes, and 1 tmRNA. Antibiotic resistance genes (glycopeptide) were detected using ABRicate v1.0.1 (5) with the ResFinder database, but no virulence genes were identified. Prophage screening with PHASTEST v1.0.1 (6) revealed one 33.9-kb phage-related sequence. Subsystem analysis through RAST v2.0 (7) showed 586 subsystems covering 31% of the genome.

The genome sequence of *S. gallolyticus* AAM02 provides a valuable resource for comparative genomics and understanding strain-specific adaptations in traditional Bangladeshi fermented milk production.

## Data Availability

This whole-genome shotgun project has been deposited at GenBank under the accession number JBSLYR000000000. The sequenced genome is publicly available under SRA, BioProject, and BioSample numbers, which are SRR36606348, PRJNA1336682, and SAMN52081389, respectively. This genome is publicly available.
